# Sexual violence against men in Brazil: underreporting, prevalence, and associated factors

**DOI:** 10.11606/s1518-8787.2023057004523

**Published:** 2023-03-30

**Authors:** Denis Gonçalves Ferreira, Maritsa Carla de Bortoli, Paula Pexe-Machado, Gustavo Santa Roza Saggese, Maria Amelia Veras

**Affiliations:** I Faculdade de Ciências Médicas Santa Casa de São Paulo Departamento de Saúde Coletiva São Paulo SP Brasil Faculdade de Ciências Médicas da Santa Casa de São Paulo. Departamento de Saúde Coletiva. São Paulo, SP, Brasil; II Secretaria de Saúde de São Paulo Instituto de Saúde São Paulo SP Brasil Secretaria de Saúde de São Paulo. Instituto de Saúde. São Paulo, SP, Brasil; III Universidade Federal do Mato Grosso Faculdade de Medicina Programa de Pós-Graduação em Ciências da Saúde Cuiabá MT Brasil Universidade Federal do Mato Grosso. Faculdade de Medicina. Programa de Pós-Graduação em Ciências da Saúde. Cuiabá, MT, Brasil

**Keywords:** Men, Crime Victims, Sex Offenses, Domestic Violence, Underregistration, Review

## Abstract

**OBJECTIVES:**

Identifying and mapping the literature regarding sexual violence against Brazilian boys and men, as well as describing its underreporting, prevalence, and associated factors.

**METHODS:**

We conducted a scoping review by searching PubMed, Biblioteca Digital Brasileira de Teses e Dissertações, Biblioteca Virtual em Saúde, Scopus, and Web of Science databases. The inclusion criteria were: (a) surveys including data on sexual violence; (b) inclusion of boys or men as victims of sexual violence; (c) presenting statistical data on prevalence, underreporting, and factors associated with sexual violence among Brazilian boys and men.

**RESULTS:**

We found a total of 1,481 papers. Ultimately, 53 were included and had their data extracted. Most studies are quantitative in nature (n = 48). The total number of participants across studies was 1,416,480 and the prevalence of sexual violence ranged from 0.1% to 71%. It is important to note that underreporting statistical data was cited in several studies. The group with the highest prevalences was men who have sex with men and those with sexual dysfunctions. Increased tendency to drug use, social isolation, unprotected anal sex, suicidal ideation, sexual dysfunction, and post-traumatic stress disorder were statistically significant predictors for having experienced sexual violence.

**CONCLUSIONS:**

Despite the prevalence of sexual violence being high against Brazilian boys and men, this area of is surprisingly understudied and there are few studies with this exclusive scope. Social cultural issues, such as sexism, contribute to the underreporting of sexual violence. Additionally, we identified issues related to mental, sexual and reproductive health to be associated with sexual violence. Based on our findings, we recommend the implementation and development of a structural infrastructure aimed at supporting boys and men who are victims of sexual violence, and preventing negative outcomes for this affected group.

## INTRODUCTION

Sexual violence against boys and men is often neglected by various social sectors, resulting in an evidence gap around the topic1^,2^. The available information on sexual violence is mostly related to female victims, especially female children and adolescents^3^.

From an analysis of the notification data from the *Sistema de Informação de Agravos de Notificação* (Sinan), between 2009 and 2013, it was possible to observe an increase in notifications of 291.92% of sexual violence against boys and men. In 2013, cases against this population accounted for 12.58% of all reported cases^4^. Data from an epidemiological bulletin from the Ministry of Health, between 2011 and 2017, show that the proportion of total notifications of this kind of violence against male children was 25.8% and against adolescents, 7.6%^5^.

The Brazilian Public Security Forum, a non-governmental organization (NGO), systematizes, through the Public Security Yearbook, all occurrences registered in the police stations and reported to the State Public Security Secretariats. According to the analysis of this data, published in 2019, sexual violence against boys and men accounted for 14.3% of all cases reported to the police^6^. Disque Direitos Humanos, in turn, reported in an annual report, also published in 2019, 18% of all reported cases^7^.

A review of the sexual violence literature in Brazil identified a range of prevalence among men from 1% to 35%^8^. According to the author, although the variation is large, the included studies indicate a higher occurrence than presented in the official statistics of the reporting systems. Among the 40 papers included in this review, only 14 included data on the prevalence of this violence in the male population.

In an analysis of the Public Security Yearbook data, the authors state that sexual crimes are among the least reported, which can be attributed to factors such as fear of the aggressor, judgments, or guilt^9^. A study conducted with men and women from a representative sample of the urban population of São Paulo concluded that experiences of sexual violence are more difficult to report when compared to other aggressions^10^. However, because of the way men are raised in a patriarchal society, it may be more difficult for them to talk about victimization experiences, producing a major problem in the reporting of rapes against men and boys^2^.

Aside from the prevalence of sexual violence against men in Brazil being often neglected and under-reported when compared to the violence suffered by women, the characteristics of these rapes are also worthy of further investigation. In this sense, this paper aims to identify and map in the scientific literature the studies dealing with sexual violence against Brazilian boys and men in the period between 2015 and 2020, seeking to obtain data on prevalence, underreporting, and factors associated with sexual violence.

## METHODS

We developed a scoping review, according to the methodology proposed by the Joanna Briggs Institute^11^. Scoping review is a form of evidence synthesis that employs a systematic method to map all scientific literature on a given subject, such as key concepts, study characteristics, specific data according to the study objectives, and evidence gaps^12^.

### Search Process

To construct the research question, the acronym PCC was used: *population* (boys aged 0 to 18 years and men, over 18 years), *concept* (victims of sexual violence) and *context* (studies conducted with Brazilian boys or men)^13^, resulting in the research question: “What are the data on underreporting, prevalence and factors associated with sexual violence against Brazilian boys and men published between 2015 and 2020?”.

Searches were conducted on July 27, 2020, in the PubMed, Biblioteca Digital Brasileira de Teses e Dissertações (BDTD), Biblioteca Virtual em Saúde (BVS), Scopus and Web of Science databases, and in all of them using the following descriptors: Sexual Violence OR Violência Sexual OR Abuso Sexual OR Sexual Abuse OR Boys AND Men OR Meninos OR Homens AND Brazil OR Brasil.

### Inclusion and Exclusion Criteria

The type of studies selected to conduct this review included peer-reviewed articles, dissertations, and theses. The inclusion criteria were: (a) surveys including data on sexual violence; (b) inclusion of boys or men as victims of sexual violence; (c) presenting data on prevalence, underreporting, and factors associated with sexual violence among Brazilian boys and men. We excluded studies that did not present data stratified by sex, that did not have men as victims, that did not include Brazilian boys or men as victims, and that focused on other forms of violence.

### Data Screening and Extraction

The database searches yielded 1,481 papers. After removing duplicates and articles that were not available, the titles and abstracts of 1,458 studies were read, of which 1,371 were excluded because they did not match the inclusion criteria. In total, 87 texts were read in their entirety for eligibility, of which 34 were excluded: nine for not presenting data stratified by sex, nine in which men appeared only as sexual aggressors and not as victims, eight for not presenting data on Brazilian men, and eight for presenting forms of violence other than sexual (supplementary table^a^). In total, 53 papers were included in this review.

The data from the included articles were populated in a spreadsheet including: year of publication; location; type of publication; authors’ names; type of design; study objective; participants; data collection method; main findings; and prevalence of sexual violence.

Figure presents the flow chart of study selection and eligibility. The selection, eligibility, and extraction processes were performed by one researcher (DFG) and checked by another researcher (PPM).

**Figure:**
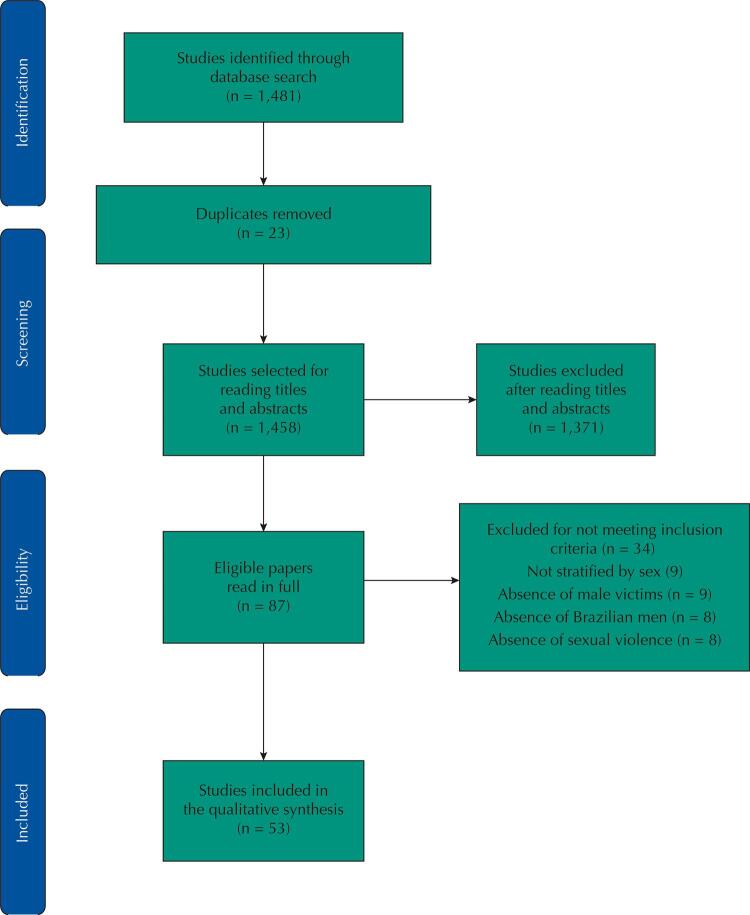
Prisma Diagram of research on sexual violence against Brazilian boys and men between 2015 and 2020.

As is the practice in scoping reviews, the results will be presented starting with the characteristics of the studies and then presenting the main findings regarding the study objectives.

## RESULTS

A total of 53 studies were included, most of them quantitative in approach (n = 48), 38 cross-sectional studies^14^, one exploratory descriptive^51^, three longitudinal^52^, one observational time-series ^4^ and one case series^55^. The remaining papers consisted of: four qualitative^56^, two literature reviews^8,20^, two intervention studies^61,62^ and one case study^63^. Among the 53 papers reviewed, 13 were conducted with secondary data^4,28,30,39^. The total number of participants was 2,831,581, of which 1,416,480 were boys or men. The research with the smallest sample size was a case study (n = 1) and the one with the largest was a study that used data from the 2015 National School Health Survey (n = 1,248,581).

The sampling techniques of the selected papers include representative population samples^10,14,21,22,25,29,46^, Respondent-driven sampling (RDS)^15,26,45^, convenience sampling^23,43,47,50,65^, birth cohort^54,53^ and multistage conglomerate survey^49^. Eight other studies did not collect primary data, using secondary data or testimony from other research^16,23,28,30,31,48,51,59^.

### Objectives of the Studies

Due to the diversity of methods used, we chose to categorize the objectives of the studies into: describing and/or analyzing factors associated with sexual violence against men (n = 23)^15,16,18,19^; understanding the phenomenon of sexual violence against this population and its impacts (n = 17)^20,31,35^; estimating the prevalence and incidence of such violence (n = 10)^4,8,14,17,23,26,37,44,53^; analyzing the perceptions of health professionals (n = 1)^56^; and evaluating intervention processes to reduce violence (n = 2)^61,62^.

Of the studies on factors associated with violence, only 14 focused on sexual violence as the main objective and, based on this, identified the factors associated with it ^18,19,22,25^. Among these papers, which focused on sexual violence and associated factors, only two exclusively discussed violence against boys and men^27,45^.

The papers included in the category “understanding the phenomenon of sexual violence against boys and men and its impacts” were studies conducted to explain the dynamics of sexual violence and its impacts. Mostly, these studies do not describe the characteristics of the victim, the aggressor, the type of violence, and the frequency. Of these surveys, only nine considered exclusively boys and men^37^.

### Ways to Identify Sexual Violence

A number of ways to identify sexual violence have been used, ranging from surveys that included questions about forced sex to instruments validated for this purpose. Among the 53 studies reviewed, only 27 reported how they investigated the presence of sexual violence. Most asked whether participants had ever been victims of forced sex in their lives (n = 11)^14,15,17,18,22,26,29,31,45,46^. Nine studies used validated instruments, such as: Assessment of childhood trauma^35^, Sexual Experiences Survey (SES-SFV)^42^, Student Alienation and Trauma Survey - R (SATS-R), Adverse childhood experiences (ACE)^50^, National Institute of Child Health and Human Development (NICHD)^57^ and Childhood Trauma Questionnaire (CTQ)^43,54^. Some studies used secondary data, mostly information from medical records of victims of sexual violence, thus they do not include screening questions.

### Participants

Most of the studies in this review did not include exclusively boys or men (n = 36). Among the studies not focused entirely on boys or men, 14 of them include children and adolescents of both sexes^14,20,28^, eight include adult women and men^8,10,19,21,25,35,44,62^, five include drug users^24,32,33,43,66^, two focus on patients in health services^21,52^, two focus on health professionals^36,56^, two target university students^42,47^, and one focuses on adolescents and adults^18^.

Among the studies exclusively with boys or men, five of them are with men who have sex with other men^15,16,26,34,45^, five on male children and adolescents^39,51,57,58,61^, three focus on service patients (mostly health services)^27,37,63^ and two focus on drug users^38,50^.

### Underreporting

Underreporting of sexual violence against boys and men has been a topic discussed in several papers. For the authors, the reasons that could explain the problem are: the inability of men to perceive themselves in the place of victim and confusion regarding sexual orientation, caused by the fact that most sexual assaults are provoked by other men^1,2,30,37,51,57,59^, the macho culture^1,2,51,58,59^, the greater difficulty of talking about sexual violence when compared to other forms of aggression^10^, the lack of training of the agents involved in the notification process^37^, the fear of reproduction of the abuse, of the parents’ reaction, of a family breakdown, and the fear of the aggressor’s reactions due to his threats^37^.

Since part of the work was based on secondary data, there are also questions about the accuracy of data recording. These data recording problems range from filling out errors^31^to missing important information, namely, the sex of the victim and the offender^51^.

### Prevalence of Sexual Violence

Most of the studies included in this review present data on prevalence of sexual violence (n = 36). Among the 17 studies conducted exclusively with boys or men, only eight present this information^15,16,26,27,34,38,45,50^.

The lowest prevalence described was 0.1% in a study carried out with a representative sample of the urban population of the city of São Paulo, which counted on information from 5,037 people, of whom 2,187 were men^10^. The highest prevalence, on the other hand, was 71%, presented in a study also carried out in São Paulo with 80 men who presented complaints of sexual dysfunctions seen at a specialized service^27^.

The groups with the highest prevalences are men who have sex with other men (14.9–59.50%)^15,16,26,34,45^, men with sexual dysfunction (10–71.3%)^27^ and alcohol or drug users (3.5-30.8%)^20,24,32,33,38,43,50^.

The variation in the prevalence data presented from Table 1 can be explained by the way the participants were selected, as well as the manner in which they were asked about their experience of sexual violence. Broader questions, such as about sexual experience before age 13 with someone five years older or more, may reveal a higher prevalence (59%)^16^ and narrower or more specific questions may produce lower response rates in participants. In the survey with men with sexual dysfunctions, when asked about rape or attempted rape (sex with penetration) the prevalence was 10%; however, when these same men were asked about any non-consensual sexual experience before the age of 12, the prevalence increased to 71.3%^27^.

**Table 1:** Characteristics of the studies included regarding the participants, scope, questions of screening of sexual violence (SV), and prevalence of sexual violence by year of publication.

Authors	Characteristics of studies				Screening for SV	Prevalence of SV

	Population (n)	Scope	Aggressor (%)	Location		
Sabidó et al., 2015^28^	MSM (3,859)^a^	National	Acquaintances (34.6)	Not informed	Have you ever been forced into sexual intercourse?	15.9% (95%CI 14.7–17.1)
			Family members (27.7)			
			Strangers (22.8)			
			Casual Partners (8.2)			
			Intimate partners (6.8)			
			Female (7.5)			
			Both (3.7)			
Thornton and Veenema, 2015^60^	Children and adolescents (Not informed)^b^	National	Not informed	Not informed	Not specified since it uses secondary data	Childhood – 1.6%–20.97%
Rates et al., 2015^31^	Children and adolescents (8,177)^b^	National	Parents (51.5)	Victim’s home (73.6%)	Have you ever been forced into sexual intercourse?	22.70%
Oldenburg et al., 2015^34^	MSM (24,051)^a^	Regional (Latin America)	Not informed in childhood, but in adulthood the aggressor is the intimate partner	Not informed	Unwanted or forced sexual contact or sexual intercourse with a person five years older (when the respondent was 13 years old or younger) or ten years older (when the respondent was 14 to 17 years old)	59.50%
Sudbrack et al., 2015^35^	Men and women (3,257)	National (internet)	Not informed	Not informed	AFECTS Questions	-
Guimarães et al., 2016^45^	Drug users (268)	Location (Goiás/GO)	Not informed	Not informed	History of sexual violence by any stable or unstable partner in the last 12 months	30.80%
Luz et al., 2016^25^	Men and women (1,620)	Location (Rio de Janeiro/RJ and São Paulo/SP)	Not informed	Not informed	Not informed	Sexual trauma in adulthood 0.9 (95%CI: 0.5–1.7)
						Childhood
						0.4 (95%CI: 0.2–1.0)
Nunes et al., 2016^63^	Psychiatric hospital patient (1)^a^	Location (Not informed)	Not informed	Not informed	-	-
Winzer, 2016^8^	Men and women (Not informed)^b^	National	Intimate partners Someone who knew or did not know the victim	Not informed	Not specified since it uses secondary data	During the lifetime–5% to 16% Last 12 months–1% to 35%
Soares et al., 2016^53^	Adolescents (1,909)	Location (Pelotas/RS)	Not informed	Not informed	Has anyone ever tried to do sexual things to you against your will, threatening or hurting you? (CTQ Form)	Childhood–0.5% (95%CI: 0.3, 1.0)
D’Abreu e Krahé, 2016^42^	University students (263)	Location (São Paulo/SP)	Not informed	Not informed	SES-SFV Questions	T1 (13.8%)
						T2 (3.7%)
Guimarães et al., 2017^20^	Drug users (783)	Location (Goiânia/GO and Campo Grande/MS	Not informed	Not informed	Not informed	8.20%
Barros et al., 2017^21^	Men and women (2,298)	National	Not informed	Not informed	Not informed	-
Campos et al., 2017^22^	Elementary School Students (1,248,581)	National	Boyfriend/ex (25.6)	Not informed	Ever had forced sex	3.57%
			Family members (19.3)			
			Friends (19.2)			
			Parents (10.5)			
Hohendorff et al., 2017^57^	Boys who are victims of SV and psychotherapists (8)^a^	Location (Rio Grande do Sul/RS	Close figures to the victim	Victim’s or perpetrator’s home	-	-
Barros and Schraiber, 2017^23^	Men and women patients of healthcare services (775)	Location (São Paulo)	Intimate partner	Not informed	Not informed	1.6%
Oliveira, 2017^59^	Male children and adolescents (Not informed)^a,b^	Location (Belo Horizonte/MG)	Identity reference men	Victim’s or perpetrator’s home	Not informed	-
Said, 2017^51^	Children and adolescents (290)^a,b^	Location (Distrito Federal/DF)	Intrafamily offenders (55.0)	Victim’s or perpetrator’s home	Not informed since medical records data were used.	-
			Female offenders (10)			
Schäfer et al., 2017^29^	Children and adolescents (1,623)^b^	Location (Lajeado and Sapiranga/RS)	Not informed	Not informed	Have you ever been forced into sexual intercourse?	1.50%
Santos et al., 2017^40^	Children and adolescents (5,357)^b^	Location (São Paulo/SP)	Not informed	Not informed	Not informed since data from the SINAN were used.	6.90%
Melo and Garcia, 2017^41^	Adolescents (144)^b^	National	Not informed	Not informed	Not informed since data from the SINAN were used.	0.20%
Carvalho et al., 2017^49^	High School Students (Not Informed)	National	Current intimate partner (5.6) Previous intimate partner (1.9) Parents or guardians (5.4)	Not informed	“Has the person you are currently dating or have dated in the last year forced you to have sex when you didn’t want to?”; “Have you ever experienced sexual aggression from other boyfriends/girlfriends or people you’ve dated throughout your life?”; “Has your relationship with your parents/guardians ever involved any sexual experiences?”; “Have you ever experienced any sexual aggression in your school/community?”	Being forced by the current partner to have sex 5.6% Suffering sexual assault from a previous intimate partner 1.9% Having had sexual experience with parents 5.4% Suffering sexual assault at school or community 1.3%
Costa et al., 2017^55^	Children and adolescents (1,110)^b^	Location (Feira de Santana/BA)	Family or victim acquaintances” (40.9)	Victim’s home (39.4%)	Not informed since medical records data from the Child Protection Council were used.	12.60%
				In the community (37.9%)		
Silva and Barroso-Junior, 2017^48^	Children and adolescents (40)^b^	Location (Salvador/BA)	Stepfather (32.6) Uncle (20) Father (16.8) Cousin (15.8) Sibling (5.3) Grandmother (3.2) Other (6.3)	Not informed	Not informed since medical records data from the Legal Medical Institute were used.	-
Gallo et al., 2017^54^	Adolescents (2,608)	Location (Pelotas/RS)	Not informed	Not informed	Has anyone ever tried to do sexual things to you against your will, threatening or hurting you? (CTQ Form)	Childhood–0.34%
Mann and Monteiro, 2018^56^	Healthcare professionals (61)	Location (Rio de Janeiro/RJ)	Not informed	Not informed	-	-
Platt et al., 2018^28^	Pediatric hospital patients (120)^b^	Location (Florianópolis/SC)	Male (88.8)	Victim’s or perpetrator’s home (81.6%)	Not informed since medical records data were used.	-
Coêlho et al., 2018^10^	Men and women (2,187)	Location (São Paulo/SP)	Not informed	Not informed	Caresses, attempted rape (including complete sexual intercourse with penetration of fingers, objects, or genital) before the age of 18.	0.10%
Nascimento et al., 2018^61^	Adolescents in socio-educational measures and employees of the Fundação Casa (125)^a^	Location (Rio de Janeiro/RJ)	Participants had committed sexual crimes	Not informed	-	-
Madalena and Sartes, 2018^33^	Drug users (54)	Location (Zona da Mata/MG)	Not informed	Not informed	ASI 6 Questions	3.70%
Silva and Roncalli, 2018^44^	Men and women (20,031)^b^	National	Not informed	Not informed	Not informed since data from the Notifiable Diseases Information System (SINAN) were used.	8.9%
Guimaraes et al., 2018^45^	MSM (7,925)^a^	National	Not informed	Not informed	Have you ever been forced into sexual intercourse?	2009–14.9% (95%CI: 12.6%–17.1%) 2016–20.9% (95%CI: 17.8%–24.1%)
Costa et al., 2018^46^	Elementary School Students (Not Informed)^b^	National	Boyfriend/ex (26.6) Friends (21.8) Strangers (13.4) Others (13.3) Father/mother/stepfather/stepmother (11.9) Other family members (19.7)	Not informed	Have you ever been forced into sexual intercourse?	Brazil 3.7% (95%CI: 3.3–4.1) North 4.3% (95%CI: 3.6–5.0) Northeast 3.8% (95%CI: 3.3–4.3) Southeast 3.6% (95%CI: 2.8–4.4) South 3.2% (95%CI: 2.5–3.8) Midwest 4.1% (95%CI: 3.5–4.6
Gaspar and Pereira, 2018^4^	General population (Not informed)^b^	National	Friend Acquainted	Public space Home	Not informed since data from the Notifiable Diseases Information System (SINAN) were used.	2009–1.053 2013–3.074
Albuquerque and Williams, 2018^47^	University students (312)	Location (São Paulo/SP)	Not informed	Not informed	SATS-R Questions	43.80%
Vertamatti et al., 2019^30^	Children and adolescents attended in a specialized program in SV (141)^b^	Location (São Paulo/SP)	Stepfather, friends, and parents	Victim’s or perpetrator’s home	Not informed since medical records data were used.	-
Silva et al., 2019^14^	Elementary School Students (102,072)^b^	National	Not informed	Not informed	Have you ever been forced into sexual intercourse?	3.7 (95%CI: 3.3–4.1)
Edeza et al., 2019^16^	MSM (22,698)^a^	Regional (Latin America)	Not informed	Not informed	Sexual experience before 13 years old with someone five years older and sexual experience from 13 to 17 years with someone ten years older	59%
Sanchez et al., 2019^19^	Men and women (1,111)	Location (São Paulo/SP)	Strangers	Nightclubs	Have you ever experienced sexual assault, sexual harassment, forced kissing, groping, rape, or attempted rape in your lifetime?	Sexual Assault 11.7%; (95%IC: 6.7-19.6); Forced Kiss 9.7%; (95%IC: 5.9–15.3); Attempted Rape 1.4% (95%CI: 0.7–3.1); Rape 0.6% (95%CI: 0.2–3.5)
Pap, 2019^27^	Male patients of a Urology Clinic (80)^a^	Location (São Paulo/SP)	Family (31.3) Acquaintances (63.7)	Not informed	Have you ever experienced sexual assault, sexual harassment, forced kissing, groping, rape, or attempted rape in your lifetime?	Sexual abuse or attempted penetration–10%; Other forms of sexual violence–71.3%
Kato-Wallace et al., 2019^62^	Young men and adolescents (Not informed)^a^	National	Not informed	Not informed	-	-
Massaro et al., 2019^17^	Men and women (1,918)	National	Not informed	Not informed	“Have you ever been forced to have sex with someone?”	1.70%
Canfield et al., 2019^38^	Drug users (162)^a^	Multicenter (São Paulo and London)	Intimate partner	Not informed	WHO Multi-country Study on Men and Violence Questions	27.4%
Nisida et al., 2019^52^	Men and women patients of healthcare services (39)	Location (São Paulo/SP)	Not informed	Not informed	Not informed since medical records data were used.	-
Penso et al., 2019^39^	Children and adolescents (35)^a,b^	Location (Distrito Federal/DF)	Cousin, schoolmate, uncle, father, stepfather, neighbor, maid	Not informed	Not informed since medical records data were used.	-
Sanvicente-Vieira et al., 2019^43^	Drug users (797)	Location (Porto Alegre/RS)	Not informed	Not informed	CTQ and ASI-6 Questions	Sexual harassment (9.4%) Rape in adulthood (6.7%) Childhood (3.5%)
Carvalho, 2020^58^	Boys victims of SV and caregivers (6)^a,b^	Location (Campinas/SP)	Parents and stepmothers	Victim’s or perpetrator’s home	Not informed since medical records data were used.	-
Rocha et al., 2020^15^	MSM (4,129)^a^	National	Not informed	Not informed	“Have you ever been forced to have sex with someone?”	< 25 year old – 24.4%
						< 25 year old – 16.6%
Roglio et al., 2020^32^	Drug users (247)^b^	Location (Porto Alegre/RS)	Not informed	Not informed	Sexual abuse in life or childhood	23.9%
Ziliotto et al., 2020^36^	Psychologists (47)	Internet	Women (mothers with 13.9)	Victim’s or aggressor home	-	-
Conceição et al., 2020^37^	Children and adolescents in public healthcare service (35)^a,b^	Location (Brasília/DF)	Not informed	Not informed	Not informed since medical records data were used.	-
Diehl et al., 2020^18^	Adolescents and adults (1,918)	National	Not informed	Not informed	“Have you ever been forced to have sex with someone?”	1.40%

MSM: men who have sex with men; AFECTS: Affective and Emotional Composite Temperament; CTQ FORM: Childhood Trauma Questionnaire; SES-SFV: Short Form Victimization; ASI-6: Addiction Severity Index; SATS-R: Student Alienation and Trauma Survey - R.

^a^ Studies with only men

^b^ Studies with secondary data

The research that investigated sexual assaults in nightclubs in São Paulo was one of the few to include male and female people, and the prevalence of sexual violence was higher among men: 1.4% of men and 0.7% of women reported attempted rape; regarding sexual assault, the numbers were 11.7% and 11.1%, respectively^19^. Another study in which the prevalence of sexual violence was higher among boys was conducted among adolescents of both sexes, students in the second year of high school in ten Brazilian capitals. In this study, the prevalence among boys was 12.5%^49^. In the others, with people of both sexes, the prevalence of sexual violence was higher among girls and women^8,20,46,53^, as well as in the studies in which risk was estimated: girls and women were identified at higher risk of being raped than men^18,54^.

### Characteristics of Sexual Violence

Only 23 papers present information that identified the aggressor, most of them people known to the victims. Violence perpetrated by the father or stepfather was common in eight studies^22,30,31,39,46,48,58,59^. Authors who have not described who the perpetrators are point to known and/or familiar figures^4,26,27,51,57,65^. There are also studies that describe violence committed by intimate partners^8,23,38,50^. It is noteworthy that women appeared in six studies as sexual rapists^28,36,39,46,51,58^.

A minority of the studies (n = 11) indicate where the violence occurred, and almost all of them (n = 10) report that the cases occurred at the victim’s or perpetrator’s home^4,28,30,31,36,51,55,57^. Most also did not report whether the assault was repeat violence: only 12 studies provided this information4^,27,28,30,31,36,43,48,49,51,57,59^.

### Factors Associated with Sexual Violence

Several factors have been described to be associated with sexual violence. Negative mental health outcomes were the most frequently cited, including: post-traumatic stress disorder (PTSD)^25,66^; suicidal ideation^29^; drug use and social isolation^17,22,67^; and psychosis^63^. In addition to mental health issues, having unprotected anal sex^15,26^ and sexual dysfunction^27^ were also related to sexual violence.

Other forms of violence were associated with sexual violence, among them: abuse in childhood and physical violence^32^; discrimination due to sexual orientation^26^; intimate partner violence^34^; rape in adulthood^18^. In a study that analyzed the victims’ records, it was possible to identify that the duration and severity of the abuse were associated with the sexual violence suffered by boys^30^. Table 1 describes the main characteristics of the studies.

## DISCUSSION

Our study indicates a lack of research that exclusively studies sexual violence perpetrated against the adult or child male population. We also identified the underreporting of this kind of violence, an aspect cited in several studies, which may have causes in cultural norms, making it difficult for boys and men to perceive themselves as victims or even to talk about experiences that may indicate some fragility. However, prevalence studies show that sexual violence against this part of the population is a problem of great magnitude, with a significant variety of associated factors, such as negative mental health outcomes, behavioral or socialization problems, and clinical issues. Few studies had representative samples and the nature of the screening questions profoundly influenced the variation in estimated prevalences. In the surveys in which people of both genders were included, the prevalence was higher for females, corroborating the results of other studies that point to women and girls as the main victims of sexual violence^8,12,68^.

According to a study conducted in 2013 by the Institute for Applied Economic Research (IPEA), only 10% of cases of sexual violence are notified^69^ and, although researchers show an evolution of 291.92% in the notifications of this violence against the male population between 2009-2013^4^, underreporting still appears as a major problem. The reasons listed by the authors to explain this underreporting are: confusions in relation to sexual orientation, because the abuses are mostly committed by men; the sexist and patriarchal culture that makes it impossible for men to perceive themselves as victims and to talk about their emotions; fear of family breakdown; fear of the aggressor; the choice of the aggressors for very young boys, i.e., not mature enough to understand that they are being victimized; and the fear of reproduction of the abuse^1,2,30,37,51,57,59^.

Underreporting is a problem in many ways, but the fact that boys are not able to talk about their sexual trauma until much later may be associated with more severe and longer-lasting abuse^30^. In a study carried out with surveillance data, sexual violence against men and boys happened earlier when compared to girls, and the explanation for this phenomenon lies in the incapacity of the younger child to recognize the situation as violating and to break the silence^28^. This becomes even more difficult when one considers the sociocultural expectation that men are strong and resilient, generating a possible constraint for those who suffer sexual violence^6,20^.

Although there is a major problem regarding the underreporting of sexual violence against men, the prevalence data described in the studies range from 0.1% to 71%. Men who have sex with men, men with sexual dysfunctions, and drug users had higher prevalences than the other groups. In a population-based study with a representative sample from the USA, researchers found that bisexual and homosexual men were more likely to have a history of childhood sexual violence than heterosexual men^70^. Another aspect identified in the studies that make up this review is that most of them were about children and adolescents or investigated aggression in childhood. Only three studies investigated violence in adulthood, indicating a gap in evidence on sexual violence against adult men.

The National Health Survey (PNS), conducted for the second time in 2019, included questions to screen for sexual violence and pointed out that 2.5% of men aged 18 years or older participating in the survey had experienced sexual violence once in their lifetime, which represents approximately two million men^71^. In a literature review on the prevalence of sexual violence and its characteristics, the author identified prevalence ranging from 1% to 35% among males, but only 35% of the papers included in the review contained information on male victims. This corroborates the need for more studies on Brazilian men who suffer or have suffered sexual violence^2^.

The definition of sexual violence adopted by the researchers changed the prevalence results. Studies investigating all forms of sexual violence found higher prevalences; studies with more specific questions, on the other hand, showed lower prevalences. We find it difficult to choose a screening question that can be used widely, but we see the need for debate around this issue.

Regarding the factors associated with sexual violence against men and boys, this review points to a diversity of outcomes, from negative mental health outcomes (PTSD, drug use and abuse, suicidal ideation)^25,66^ to clinical problems (erectile dysfunction and premature ejaculation, and chronic pelvic pain)^27^ and behavioral issues (frequent HIV testing, unprotected anal sex, social isolation, and commercial sex)^15,17,22,26,67^. Several studies also indicate the presence of suicidal thoughts, social isolation, sexually transmitted infections (STIs), guilt, low self-esteem, psychosomatic illnesses, and physical and emotional development problems in victims^2,26,72-74^. In this sense, our results do not differ from other studies, indicating indeed a serious public health problem.

The characteristics of the violence, such as the sex of the aggressor and the place where the violence happened, were one of the points less explored by the researchers, and even so, the results indicate that in relation to the characteristics of the aggression, sexual violence suffered by men and women are similar, because the aggressors and the places have the same identifying features^8,71^.

It is important to point out that this scoping review has some limitations that should be considered when interpreting the results. Important databases were chosen to carry out the search for the studies, but it is recognized that there is a limitation in the variety of the type of material analyzed, since government documents and the proceedings of scientific events, for example, were not included.

It is not common practice in scope reviews to adopt quality assessment of the studies and statistical treatment for quantitative data, as is the case for systematic reviews. Therefore, there may be a limitation regarding the quality and scientific rigor of the studies included.

## CONCLUSIONS

In the period studied, the underreporting of sexual violence against men and boys seems to be a problem of great magnitude, which can be explained from the cultural norms surrounding the male gender. The prevalence of this violence, although largely variable, is higher than the data found in the notification systems, which may indicate the problem of underreporting in the official systems. Regarding the associated factors, there is a body of data presented indicating that men victim to sexual violence may suffer a series of consequences in several areas of their lives.

In this sense, efforts are needed for the realization of future studies with representative samples, screening questions of sexual violence that capture all forms of non-consensual sexual contact, aiming to understand the violence suffered by the male population, and with primary data, avoiding any bias in filling out the notifications. It is important that future studies are able to describe the characteristics of these assaults against men and boys, such as how long they lasted and how long the victims took to talk about what happened.

In Brazil, the Specialized Reference Centers of Social Assistance (CREAS) serves children and adolescents who are victims of rights violation, but these services are mostly used by female children. We know of only one Brazilian initiative that offers psychological care for male victims of sexual violence: *Memórias Masculinas*. Ultimately, we suggest that public policy makers should focus on developing strategies to be more inclusive of the male experience with sexual violence, and explore methods to prevent adverse effects.

## Footnotes

a Supplementary table available from: https://docs.google.com/document/d/1pEAF7l6BcZFZS1V36MvJZvE0hf99L7gx/edit?usp=sharing&ouid=114531022125527350337&rtpof=true&sd=true
