# Supplementary material for: Sexual violence against men in Brazil: underreporting, prevalence, and associated factors
Source: Rev Saude Publica. 2023 Mar 30;57:23. doi: 10.11606/s1518-8787.2023057004523 (PMC10118408; doi:10.11606/s1518-8787.2023057004523)
Supplement: Translation

# Translation: Violência sexual contra homens no Brasil: subnotificação, prevalência e fatores associados

**Authors:** Denis Gonçalves Ferreira, Maritsa Carla de Bortoli, Paula Pexe-Machado, Gustavo Santa Roza Saggese, Maria Amelia Veras

**Affiliations:** I Brasil Faculdade de Ciências Médicas da Santa Casa de São Paulo. Departamento de Saúde Coletiva. São Paulo, SP, Brasil; II Brasil Secretaria de Saúde de São Paulo. Instituto de Saúde. São Paulo, SP, Brasil; III Brasil Universidade Federal do Mato Grosso. Faculdade de Medicina. Programa de Pós-Graduação em Ciências da Saúde. Cuiabá, MT, Brasil

**Keywords:** Homens, Vítimas de Crime, Delitos Sexuais, Violência Doméstica, Sub-Registro, Revisão

## Abstract

**OBJETIVOS:**

Identificar e mapear a literatura referente à violência sexual contra meninos e homens brasileiros, bem como descrever sua subnotificação, sua prevalência e os fatores associados.

**MÉTODOS:**

Realizou-se uma revisão de escopo com buscas nas bases de dados: PubMed, Biblioteca Digital Brasileira de Teses e Dissertações, Biblioteca Virtual em Saúde, Scopus e Web of Science. Os critérios de inclusão foram: (a) pesquisas que incluíssem dados sobre violência sexual; (b) inclusão de meninos ou homens como vítimas de violência sexual; (c) apresentassem dados sobre prevalência, subnotificação e fatores associados à violência sexual entre meninos e homens brasileiros.

**RESULTADOS:**

Foram encontrados 1.481 trabalhos. No total, 53 foram incluídos e tiveram seus dados extraídos. A maioria dos estudos é de natureza quantitativa (n = 48). O total de participantes em todos os estudos foi de 1.416.480 e a prevalência de violência sexual variou de 0.1% a 71%. A subnotificação foi um aspecto citado em vários estudos. Entre os grupos com maiores prevalências estão os homens que fazem sexo com homens e com disfunções sexuais. Maior tendência ao uso de drogas, isolamento social, sexo anal desprotegido, ideação suicida, disfunções sexuais e transtorno de estresse pós-traumático foram alguns dos fatores associados.

**CONCLUSÕES:**

A violência sexual contra meninos e homens brasileiros é pouco estudada e existem poucos estudos com esse recorte exclusivo, apesar da prevalência de a violência sexual ser alta. Questões culturais, como o machismo, contribuem para a subnotificação da violência sexual. Em relação aos fatores associados, identificamos questões relacionadas à saúde mental, sexual e reprodutiva. Recomenda-se que seja estruturado acolhimento para meninos e homens vítimas de violência sexual, prevenindo ou minimizando desfechos negativos.

## INTRODUÇÃO

A violência sexual contra meninos e homens é negligenciada por diversos setores sociais, resultando em uma lacuna de evidências em torno do tema^1,2^. As informações disponíveis sobre violência sexual são majoritariamente relacionadas a vítimas do gênero feminino, em especial crianças e adolescentes do gênero feminino^3^.

A partir de uma análise dos dados de notificação do Sistema de Informação de Agravos de Notificação (Sinan), entre os anos de 2009 e 2013, foi possível observar um aumento nas notificações de 291.92% de violência sexual contra meninos e homens. Em 2013, os casos contra essa população representavam 12,58% do total de casos notificados^4^. Dados de um boletim epidemiológico do Ministério da Saúde, entre os anos de 2011 e 2017, mostram que a proporção do total de notificações dessa violência contra crianças do sexo masculino era de 25,8% e contra adolescentes, de 7,6%^5^.

O Fórum Brasileiro de Segurança Pública, uma organização não-governamental (ONG), sistematiza por meio do Anuário de Segurança Pública todas as ocorrências registradas nas delegacias e reportadas às Secretarias Estaduais de Segurança Pública. A partir da análise desses dados, publicados em 2019, é possível observar que a violência sexual contra meninos e homens representou 14,3% de todos os casos reportados à polícia^6^. O Disque Direitos Humanos, por sua vez, informou em relatório anual, também publicado em 2019, apontou que o percentual era 18% de todos os casos denunciados^7^.

Uma revisão da literatura sobre violência sexual no Brasil identificou uma variação de prevalência entre homens de 1% a 35%^8^. De acordo com a autora, embora a variação seja grande, os estudos incluídos indicam uma ocorrência mais elevada do que a apresentada nas estatísticas oficiais dos sistemas de notificação. Entre os 40 trabalhos incluídos nessa revisão apenas 14 incluíram dados sobre a prevalência dessa violência sobre a população masculina.

Em análise dos dados do Anuário de Segurança Pública, os autores afirmam que os crimes sexuais estão entre os menos notificados, o que pode ser atribuído a fatores como medo do agressor, de julgamentos ou por culpa^9^. Em estudo realizado com homens e mulheres a partir de uma amostra representativa da população urbana de São Paulo, concluiu-se que experiências de violência sexual são mais difíceis de serem reportadas quando comparadas a outras agressões^10^. Todavia, em virtude da maneira como os homens são educados na sociedade patriarcal, pode ser mais difícil para eles falar sobre experiências de vitimização, produzindo um problema importante na notificação de estupros contra homens e meninos^2^.

Além de a prevalência da violência sexual contra homens no Brasil ser pouco explorada e a notificação ser menos frequente quando comparada com as violências sofridas pelas mulheres, as características desses estupros são merecedoras de investigações mais aprofundadas. Nesse sentido, este trabalho tem por objetivo identificar e mapear na literatura científica os estudos que tratem da violência sexual contra meninos e homens brasileiros no período entre 2015 e 2020, buscando levantar dados de prevalência, subnotificação e fatores associados à violência.

## MÉTODOS

Foi desenvolvida uma revisão de escopo, conforme metodologia proposta pelo Joanna Briggs Institute^11^. A revisão de escopo é uma forma de síntese de evidências que emprega um método sistemático para mapear toda a literatura científica sobre determinado assunto, como conceitos chave, características de estudos, dados específicos de acordo com os objetivos do estudo e lacunas de evidências^12^.

### Processo de Busca

Para construção da pergunta de pesquisa, utilizou-se o acrônimo PCC, *population* (meninos de 0 a 18 anos e homens, acima de 18 anos), *concept* (vítimas de violência sexual) e *context* (estudos realizados com meninos ou homens brasileiros)^13^, resultando na pergunta de pesquisa: “Quais são os dados sobre subnotificação, prevalência e fatores associados à violência sexual contra meninos e homens brasileiros publicados entre os anos de 2015 e 2020?”.

As buscas foram realizadas em 27 de julho de 2020 nas bases de dados PubMed, Biblioteca Digital Brasileira de Teses e Dissertações (BDTD), Biblioteca Virtual em Saúde (BVS), Scopus e Web of Science, e em todas foram utilizados os seguintes descritores: Sexual Violence OR Violência Sexual OR Abuso Sexual OR Sexual Abuse OR Boys AND Men OR Meninos OR Homens AND Brazil OR Brasil.

### Critérios de Inclusão e Exclusão

O tipo de fonte de dados escolhido para realização desta revisão incluiu artigos revisados por pares, dissertações e teses. Os critérios de inclusão estabelecidos foram: (a) pesquisas que incluíssem dados sobre violência sexual; (b) inclusão de meninos ou homens como vítimas de violência sexual; (c) pesquisas que apresentassem dados sobre prevalência, subnotificação e fatores associados à violência sexual contra homens brasileiros. Foram excluídos estudos que não apresentassem os dados estratificados por sexo, que não tivessem homens como vítimas, que não incluíam meninos ou homens brasileiros como vítimas e que eram focados em outras formas de violência.

### Triagem e Extração dos Dados

A partir das buscas nas bases de dados, foram encontrados 1.481 trabalhos. Após a remoção das duplicadas e artigos que não estavam disponíveis, foi realizada a leitura de títulos e resumos de 1.458 estudos, dos quais 1.371 foram excluídos, por não corresponderem aos critérios de inclusão. No total, 87 textos foram lidos na íntegra para elegibilidade, dos quais 34 foram excluídos: nove por não apresentarem os dados estratificados por sexo, nove em que os homens apareciam apenas como agressores sexuais e não vítimas, oito por não apresentarem dados sobre homens brasileiros, e oito por apresentarem outras formas de violência que não a sexual (tabela suplementar^a^). No total, 53 trabalhos foram incluídos nesta revisão.

Os dados dos artigos incluídos foram extraídos em uma planilha elaborada especificamente para esse fim, que incluía: ano de publicação; local; tipo de publicação; nome dos autores; tipo de delineamento; objetivo do estudo; participantes; método de coleta de dados; principais achados; e prevalência de violência sexual.

A Figura apresenta o fluxograma de seleção e elegibilidade dos estudos. Os processos de seleção, elegibilidade e extração foram realizados por um pesquisador (DFG) e checados por outra pesquisadora (PPM).

**Figura:** Diagrama Prisma de pesquisas sobre violência sexual contra meninos e homens brasileiros entre 2015 e 2020.

Como é prática em revisões de escopo, os resultados serão apresentados a partir das características dos estudos e posterior apresentação dos principais resultados referentes aos objetivos do estudo.

## RESULTADOS

Um total de 53 estudos foram incluídos, a maioria de abordagem quantitativa (n = 48), 38 estudos transversais^14^, um descritivo exploratório^51^, três longitudinais^52^, um observacional de série temporal^4^ e uma série de casos^55^. Os outros trabalhos consistiram em: quatro qualitativos^56^, duas revisões de literatura^8,20^, dois estudos de intervenção^61,62^ e um de estudo de caso^63^. Entre os 53 trabalhos analisados, 13 foram realizados com dados secundários^4,28,30,39^. O total de participantes foi de 2.831.581, desses, 1.416.480 eram meninos ou homens. A pesquisa com menor tamanho de amostra foi um estudo de caso (n = 1) e a com maior foi um estudo que utilizou dados da *Pesquisa Nacional de Saúde do Escolar*, de 2015 (n = 1.248.581).

As técnicas de amostragem dos trabalhos selecionados incluem amostras representativas de populações^10,14,21,22,25,29,46^, *Respondent-driven sampling* (RDS)^15,26,45^, amostra por conveniência^23,43,47,50,65,^, coorte de nascimento^54,53^ e inquérito por conglomerado multiestágio^49^. Outros oito estudos não coletaram dados primários, fazendo uso de dados secundários ou depoimentos de outras pesquisas^16,23,28,30,31,48,51,59^.

### Objetivo dos Estudos

Devido à diversidade de métodos utilizados, optou-se por categorizar os objetivos dos estudos em: descrever e/ou analisar fatores associados à violência sexual contra homens (n = 23)^15,16,18,19^; compreender o fenômeno da violência sexual contra essa população e seus impactos (n = 17)^20,31,35^; estimar a prevalência e a incidência de dessa violência (n = 10)^4,8,14,17,23,26,37,44,53^; analisar as percepções de profissionais da saúde (n = 1)^56^; e avaliar processos de intervenção para redução da violência (n = 2)^61,62^.

Dos estudos sobre os fatores associados à violência, apenas 14 focalizaram a violência sexual como objetivo principal e, a partir disso, identificaram os fatores a ela associados^18,19,22,25^. Entre esses trabalhos, que tinham como objetivo central a violência sexual e os fatores associados, apenas dois discutiram exclusivamente a violência praticada contra meninos e homens^27,45^.

As pesquisas que foram incluídas na categoria “compreender o fenômeno da violência sexual contra meninos e homens e seus impactos” eram estudos realizados basicamente para explicar a dinâmica da violência sexual e os impactos que pode produzir. Não são, majoritariamente, trabalhos que descrevem as características da vítima, do(a) agressor(a), do tipo de violência e da frequência. Dessas pesquisas, apenas nove trabalharam exclusivamente com meninos e homens^37^.

### Formas de Identificar a Violência Sexual

Foram utilizadas algumas formas de identificar a violência sexual, desde pesquisas que incluíram perguntas sobre sexo forçado até instrumentos validados para esse objetivo. Entre os 53 estudos analisados, apenas 27 informam como investigaram a presença da violência sexual. A maioria questionou se os participantes foram vítimas de sexo forçado alguma vez na vida (n = 11)^14,15,17,18,22,26,29,31,45,46^. Nove trabalhos utilizaram instrumentos validados, como: *Assessment of childhood trauma*^35^, *Sexual Experiences Survey* (SES-SFV)^42^, *Student Alienation and Trauma Survey – R* (SATS-R), *Adverse childhood experiences* (ACE)^50^, *National Institute of Child Health and Human Development* (NICHD)^57^ e *Childhood Trauma Questionnaire* (CTQ)^43,54^. Alguns estudos recorreram a dados secundários, majoritariamente informações de prontuários de vítimas de violência sexual, portanto, não há perguntas de rastreio descrita neles.

### Participantes

A maioria dos estudos que compõem esta revisão não trabalhou exclusivamente com meninos ou homens (n = 36). Dentre os estudos não exclusivos com meninos ou homens, 14 deles são com crianças e adolescentes de ambos os sexos^14,20,28^, oito com mulheres e homens adultos^8,10,19,21,25,35,44,62^, cinco com usuários de drogas^24,32,33,43,66^, dois com pacientes em serviços de saúde^21,52^, dois com profissionais de saúde^36,56^, dois com estudantes universitários^42,47^ e um com adolescentes e adultos^18^.

Entre os estudos exclusivos com meninos ou homens, cinco deles são com homens que fazem sexo com outros homens^15,16,26,34,45^, outros cinco fazem o recorte em crianças e adolescentes do sexo masculino^39,51,57,58,61^, três focalizam pacientes de serviços (majoritariamente serviços de saúde)^27,37,63^ e dois enquadram usuários de drogas^38,50^.

### Subnotificação

A questão da subnotificação da violência sexual contra meninos e homens foi um tema discutido em diversos trabalhos. Para os autores, as razões que poderiam explicar o problema são: a incapacidade do homem de se perceber no lugar de vítima e a confusão em relação à orientação sexual, provocada pelo fato de a maioria das agressões sexuais ser provocada por outros homens^1,2,30,37,51,57,59^, a cultura machista^1,2,51,58,59^, a maior dificuldade de falar sobre violência sexual quando comparada com outras formas de agressão^10^, a falta de treinamento dos agentes envolvidos no processo de notificação^37^, o medo da reprodução do abuso, da reação dos pais, de uma ruptura familiar e o medo das reações do agressor em virtude de suas ameaças^37^.

Como parte dos trabalhos foi realizada baseando-se em dados secundários, há também questões acerca da qualidade do preenchimento das fichas de notificação. Esses problemas variam desde erro no preenchimento^31^ até a falta de informações importantes, como o sexo da vítima e do agressor^51^.

### Prevalência de Violência Sexual

A maioria dos estudos incluídos nesta revisão apresenta dados sobre prevalência de violência sexual (n = 36). Entre os 17 estudos realizados exclusivamente com meninos ou homens, apenas oito apresentam essas informações^15,16,26,27,34,38,45,50^.

A menor prevalência descrita foi de 0,1% em estudo realizado com amostra representativa da população urbana da cidade de São Paulo, que contou com informações de 5.037 pessoas, em que 2.187 eram homens^10^. Já a maior prevalência foi de 71%, apresentada em um estudo realizado também na cidade de São Paulo com 80 homens que apresentavam queixa de disfunções sexuais atendidos em um serviço especializado^27^.

Os grupos com prevalências mais altas são os de homens que fazem sexo com outros homens (14.9–59,50%)^15,16,26,34,45^, homens com disfunções sexuais (10–71,3%)^27^ e usuários de álcool ou drogas (3.5–30,8%)^20,24,32,33,38,43,50^.

A variação dos dados de prevalência apresentados da Tabela 1 pode ser explicada pela forma como foram selecionados os participantes, assim como a maneira com que foram questionados sobre a experiência de violência sexual. Perguntas mais amplas, como sobre experiência sexual antes dos 13 anos com alguém cinco anos mais velho ou mais, podem revelar prevalências mais altas (59%)^16^ e perguntas mais fechadas ou específicas podem produzir menor índice de resposta nos participantes. Na pesquisa com homens com disfunções sexuais, ao serem questionados sobre estupro ou tentativa de estupro (sexo com penetração) a prevalência foi de 10%, todavia, quando esses mesmos homens foram questionados sobre qualquer experiência sexual não consentida antes dos 12 anos, a prevalência aumenta para 71,3%^27^.

**Tabela 1:** Características dos estudos incluídos em relação aos participantes, abrangência, perguntas de rastreio da violência sexual (VS) e prevalência de violência sexual por ano de publicação.

Autores	Características dos estudos				Rastreio da VS	Prevalência de VS

	População (n)	Abrangência	Agressor(a) (%)	Local		
Sabidó et al., 2015^28^	HSH (3.859)^a^	Nacional	Conhecidos (34,6)	Não informado	Alguma vez na vida você foi forçado a ter relação sexual?	15,9% (95%CI 14,7–17,1)
			Familiares (27,7)			
			Estranhos (22,8)			
			Parceiros casuais (8,2)			
			Parceiros íntimos (6,8)			
			Sexo feminino (7,5)			
			Ambos (3,7)			
Thornton e Veenema, 2015^60^	Crianças e adolescentes (Não informado)^b^	Nacional	Não informado	Não informado	Não especificado por que usa dados secundários	Infância – 1,6%–20,97%
Rates et al., 2015^31^	Crianças e adolescentes (8.177)^b^	Nacional	Pais (51,5)	Residência da vítima (73.6%)	Alguma vez na vida você foi forçado(a) a ter relação sexual?	22,70%
Oldenburg et al., 2015^34^	HSH (24.051)^a^	Regional (América Latina)	Não informado na infância, mas na idade adulta é o parceiro íntimo	Não informado	Contato sexual indesejado ou forçado ou relações sexuais com uma pessoa 5 anos mais velha (quando o respondente tinha 13 anos de idade ou menos) ou 10 anos mais velho (quando o respondente tinha entre 14-17 anos)	59,50%
Sudbrack et al., 2015^35^	Homens e mulheres (3.257)	Nacional (internet)	Não informado	Não informado	Perguntas do *AFECTS*	-
Guimarães et al., 2016^45^	Usuários de drogas (268)	Local (Goiás/GO)	Não informado	Não informado	Histórico de violência sexual por algum (a) parceiro (a) estável ou não nos últimos 12 meses	30,80%
Luz et al., 2016^25^	Homens e mulheres (1.620)	Local (Rio de Janeiro/RJ e São Paulo/SP)	Não informado	Não informado	Não informado	Trauma sexual na idade adulta 0,9 (95%CI: 0,5–1,7)
						Infância
						0,4 (95%CI: 0,2–1,0)
Nunes et al., 2016^63^	Paciente de hospital Psiquiátrico (1)^a^	Local (Não informado)	Não informado	Não informado	-	-
Winzer, 2016^8^	Homens em mulheres (Não informado)^b^	Nacional	Parceiros/as íntimos Alguém que conhecia ou não a vítima	Não informado	Não especificado por que usa dados secundários	Durante a vida–5% a 16% Últimos 12 meses–1% a 35%
Soares et al., 2016^53^	Adolescentes (1.909)	Local (Pelotas/RS)	Não informado	Não informado	Alguém já tentou fazer coisas sexuais com você contra sua vontade, ameaçando ou machucando-o? *(CTQ Form)*	Infância–0,5% (95%CI: 0,3, 1,0)
D’Abreu e Krahé, 2016^42^	Estudantes universitários (263)	Local (São Paulo/SP)	Não informado	Não informado	Perguntas do *SES-SFV*	T1 (13,8%)
						T2 (3,7%)
Guimarães et al., 2017^20^	Usuários de drogas (783)	Local (Goiânia/GO e Campo Grande/MS	Não informado	Não informado	Não informado	8,20%
Barros et al., 2017^21^	Homens e mulheres (2.298)	Nacional	Não informado	Não informado	Não informado	-
Campos et al., 2017^22^	Estudantes do Ensino Fundamental (1.248.581)	Nacional	Namorado/ex (25,6)	Não informado	Sexo forçado alguma vez na vida	3,57%
			Familiares (19,3)			
			Amigos (19,2)			
			Pais (10,5)			
Hohendorff et al., 2017^57^	Meninos vítimas de VS e psicoterapeutas (8)^a^	Local (Rio Grande do Sul/RS	Figuras próximas da vítima	Residência da vítima ou do autor	-	-
Barros e Schraiber, 2017^23^	Homens e mulheres pacientes de serviços de saúde (775)	Local (São Paulo)	Parceiro íntimo	Não informado	Não informado	1,6%
Oliveira, 2017^59^	Crianças e adolescentes do sexo masculino (Não informado)^a,b^	Local (Belo Horizonte/MG)	Homens referenciais identitários	Residência da vítima ou do autor	Não informado	-
Said, 2017^51^	Crianças e adolescentes (290)^a,b^	Local (Distrito Federal/DF)	Ofensores intrafamiliares (55,0)	Residência da vítima ou do autor	Não informado por que usou dados de prontuários.	-
			Ofensoras do sexo feminino (10)			
Schäfer et al., 2017^29^	Crianças e adolescentes (1.623)^b^	Local (Lajeado e Sapiranga/RS)	Não informado	Não informado	Alguma vez na vida você foi forçado(a) a ter relação sexual?	1,50%
Santos et al., 2017^40^	Crianças e adolescentes (5.357)^b^	Local (São Paulo/SP)	Não informado	Não informado	Não informado por que usou dados do sistema de notificação (Sinan)	6,90%
Melo e Garcia, 2017^41^	Adolescentes (144)^b^	Nacional	Não informado	Não informado	Não informado por que usou dados do sistema de notificação (Sinan)	0,20%
Carvalho et al., 2017^49^	Estudantes do Ensino Médio (Não informado)	Nacional	Atual parceiro íntimo (5,6) Parceiro afetivo anterior (1,9) Pais ou responsáveis (5,4)	Não informado	“a pessoa com quem ‘fica’ ou namora atualmente ou no último ano forçou você a fazer sexo quando não queria?”; “já sofreu agressão sexual de outros(as) namorados(as) ou pessoa com quem “ficou” ao longo da vida?”; “a sua relação com seus pais/responsáveis já envolveu alguma experiência sexual?”; “você já sofreu alguma agressão sexual na sua escola/comunidade?”	Ser forçado pelo atual parceiro a fazer sexo 5,6% Sofrer agressão sexual de parceiro afetivo anterior 1,9% Ter passado por experiência sexual com pais 5,4% Sofrer agressão sexual na escola ou comunidade 1,3%
Costa et al., 2017^55^	Crianças e adolescentes (1.110)^b^	Local (Feira de Santana/BA)	Conhecidos da família ou da vítima” (40,9)	Residência da vítima (39,4%)	Não informado por que usou dados de prontuários do Conselho Tutelar	12,60%
				Na comunidade (37,9%)		
Silva e Barroso-Junior, 2017^48^	Crianças e adolescentes (40)^b^	Local (Salvador/BA)	Padrasto (32,6) Tio (20) Pai (16,8) Primo (15,8) Irmão (5,3) Avó (3,2) Outro (6,3)	Não informado	Não informado por que usou dados de prontuários do Instituto Médico Legal	-
Gallo et al., 2017^54^	Adolescentes (2.608)	Local (Pelotas/RS)	Não informado	Não informado	Alguém já tentou fazer coisas sexuais com você contra sua vontade, ameaçando ou machucando-o? (*CTQ Form*)	Infância–0,34%
Mann e Monteiro, 2018^56^	Profissionais de saúde (61)	Local (Rio de Janeiro/RJ)	Não informado	Não informado	-	-
Platt et al., 2018^28^	Pacientes de hospital pediátrico (120)^b^	Local (Florianópolis/SC)	Sexo masculino (88,8)	Residência da vítima ou do autor (81.6%)	Não informado por que usou dados de prontuário	-
Coêlho et al., 2018^10^	Homens e mulheres (2.187)	Local (São Paulo/SP)	Não informado	Não informado	Carícias, tentativa de estupro (incluindo relação sexual completa com penetração de dedos, objetos ou genital) antes dos 18 anos.	0,10%
Nascimento et al., 2018^61^	Adolescentes em medida sociodeducativa e funcionários da Fundação Casa (125)^a^	Local (Rio de Janeiro/RJ)	Os participantes haviam cometido crimes sexuais	Não informado	-	-
Madalena e Sartes, 2018^33^	Usuários de drogas (54)	Local (Zona da Mata/MG)	Não informado	Não informado	Perguntas do *ASI 6*	3,70%
Silva e Roncalli, 2018^44^	Homens e mulheres (20.031)^b^	Nacional	Não informado	Não informado	Não informado por que usou dados do sistema de notificação (*Sinan*)	8,9%
Guimaraes et al., 2018^45^	HSH (7925)^a^	Nacional	Não informado	Não informado	Alguma vez na vida você foi forçado(a) a ter relação sexual?	2009–14,9% (95%CI: 12,6%–17,1%) 2016–20,9% (95%CI: 17,8%–24,1%)
Costa et al., 2018^46^	Estudantes do Ensino Fundamental (Não informado)^b^	Nacional	Namorado/ex namorado (26,6) Amigos (21,8) Desconhecido (13,4) Outros (13,3) Pai/mãe/ padrasto/madrasta (11,9) Outros familiares (19,7)	Não informado	Alguma vez na vida você foi forçado(a) a ter relação sexual?	Brasil 3,7% (95%CI: 3,3–4,1) Norte 4,3% (95%CI: 3,6–5,0) Nordeste 3,8% (95%CI: 3,3–4,3) Sudeste 3,6% (95%CI: 2,8–4,4) Sul 3,2% (95%CI: 2,5-3,8) Centro-oeste 4,1% (95%CI: 3,5-4,6)
Gaspar e Pereira, 2018^4^	População geral (Não informado)^b^	Nacional	Amigo Conhecido	Via pública Residência	Não informado por que usou dados do sistema de notificação (Sinan)	2009–1,053 2013–3,074
Albuquerque e Williams, 2018^47^	Estudantes universitários (312)	Local (São Paulo/SP)	Não informado	Não informado	Perguntas do *SATS-R*	43,80%
Vertamatti et al., 2019^30^	Crianças e adolescentes atendimentos num programa especializado em VS (141)^b^	Local (São Paulo/SP)	Padrasto, amigos e pais	Residência da vítima ou do autor	Não informado por que usou dados de prontuários	-
Silva et al., 2019^14^	Estudantes do Ensino Fundamental (102.072)^b^	Nacional	Não informado	Não informado	Alguma vez na vida você foi forçado(a) a ter relação sexual?	3,7 (95%CI: 3,3–4,1)
Edeza et al., 2019^16^	HSH (22.698)^a^	Regional (América Latina)	Não informado	Não informado	Experiência sexual antes dos 13 anos com alguém 5 anos mais velho e experiência sexual entre os 13-17 anos com alguém 10 anos mais velho	59%
Sanchez et al., 2019^19^	Homens e mulheres (1.111)	Local (São Paulo/SP)	Desconhecidos	Casas noturnas	Alguma vez na vida você sofreu agressão sexual, assédio sexual, beijo forçado, passada de mão, estupro, tentativa de estupro?	Agressão Sexual 11,7%; (95%IC: 6,7-19,6); Beijo Forçado 9,7%; (95%IC: 5,9–15,3); Tentativa de Estupro 1,4% (95%IC: 0,7–3,1); Estupro 0,6% (95%IC: 0,2–3,5)
Pap, 2019^27^	Homens pacientes da uma Clínica de Urologia (80)^a^	Local (São Paulo/SP)	Família (31,3) Conhecidos (63,7)	Não informado	Alguma vez na vida você sofreu agressão sexual, assédio sexual, beijo forçado, passada de mão, estupro, tentativa de estupro?	Abuso sexual ou tentativa de penetração–10%; Outras formas de violência sexual–71,3%
Kato-Wallace et al., 2019^62^	Homens jovens e adolescentes (Não informado)^a^	Nacional	Não informado	Não informado	-	-
Massaro et al., 2019^17^	Homens e mulheres (1.918)	Nacional	Não informado	Não informado	“Você já foi forçado(a) a ter relações sexuais com alguém?”	1,70%
Canfield et al., 2019^38^	Usuários de drogas (162)^a^	Multicêntrico (São Paulo e Londres)	Parceiro íntimo	Não informado	Perguntas do *WHO Multi-country Study on Men and Violence*	27,4%
Nisida et al., 2019^52^	Homens e Mulheres pacientes de serviços de saúde (39)	Local (São Paulo/SP)	Não informado	Não informado	Não informado por que usou dados de prontuários	-
Penso et al., 2019^39^	Crianças e adolescentes (35)^a,b^	Local (Distrito Federal/DF)	Primo, colega de escola, tio(a), pai, padrasto, vizinho, empregada	Não informado	Não informado por que usou dados de prontuários	-
Sanvicente-Vieira et al., 2019^43^	Usuários de drogas (797)	Local (Porto Alegre/RS)	Não informado	Não informado	Perguntas do *CTQ* e *ASI-6*	Assédio sexual (9,4%) Estupro na idade adulta (6,7%) Infância (3,5%)
Carvalho, 2020^58^	Meninos vítimas de VS e cuidadores (6)^a,b^	Local (Campinas/SP)	Pais e madrastas	Residência da vítima ou do autor	Não informado por que usou dados de prontuários	-
Rocha et al., 2020^15^	HSH (4.129)^a^	Nacional	Não informado	Não informado	Você já foi forçado a ter relações sexuais com alguém?	< de 25 anos – 24,4%
						< de 25 anos – 16,6%
Roglio et al., 2020^32^	Usuários de drogas (247)^b^	Local (Porto Alegre/RS)	Não informado	Não informado	Abuso sexual na vida ou na infância	23,9%
Ziliotto et al., 2020^36^	Psicólogos(as) (47)	Internet	Mulheres (mães com 13,9)	Residência da vítima ou da autora	-	-
Conceição et al., 2020^37^	Crianças e adolescentes atendidas em serviço de saúde pública (35)^a,b^	Local (Brasília/DF)	Não informado	Não informado	Não informado por que usou dados de prontuários	-
Diehl et al., 2020^18^	Adolescentes e adultos (1.918)	Nacional	Não informado	Não informado	Você já foi forçado a ter relações sexuais com alguém?	1,40%

HSH: homem que faz sexo com outro homem; AFECTS: Affective and Emotional Composite Temperament; CTQ FORM: Childhood Trauma Questionnaire; SES-SFV: Short Form Victimization; ASI-6: Addiction Severity Index; SATS-R: Student Alienation and Trauma Survey – R.

^a^ Estudos exclusivos com homens

^b^ Estudos com dados secundários

A pesquisa que investigou agressões sexuais em casas noturnas de São Paulo foi uma das únicas que incluiu pessoas do sexo masculino e feminino, e a prevalência de violência sexual foi maior entre os homens: 1,4% dos homens e 0,7% das mulheres relataram tentativa de estupro; com relação à agressão sexual, os números foram de 11,7% e 11,1%, respectivamente^19^. Outro estudo em que a prevalência de violência sexual foi maior entre os meninos foi realizado entre adolescentes de ambos os sexos, estudantes do segundo ano do Ensino Médio de dez capitais brasileiras. Nesse estudo, a prevalência entre os meninos foi de 12,5%^49^. Nos demais, com pessoas de ambos os sexos, a prevalência de violência sexual foi maior entre as meninas e mulheres^8,20,46,53^, assim como nos estudos em que se estimou o risco: as meninas e mulheres foram identificadas com maior risco de serem violentadas do que os homens^18,54^.

### Características da Violência Sexual

Apenas 23 trabalhos apresentam informações que identificavam o(a) agressor(a), a maioria deles(as) pessoas conhecidas das vítimas. Violência perpetrada pelo pai ou padrasto foi comum em oito estudos^22,30,31,39,46,48,58,59^. Autores que não descreveram quem são os(as) perpetradores apontam para figuras conhecidas e/ou familiares^4,26,27,51,57,65^. Há ainda estudos que descrevem violências cometidas por parceiros(as) íntimos(as)^8,23,38,50^. Destaca-se que as mulheres apareceram em seis estudos como violadoras sexuais^28,36,39,46,51,58^.

Uma minoria dos estudos (n = 11) indica onde as violências aconteceram e quase todos (n=10) informam que os casos ocorreram na casa da própria vítima ou do agressor(a)^4,28,30,31,36,51,55,57^. A maioria também não informou se a agressão se tratava de violência de repetição: apenas 12 estudos apresentaram essa informação^4,27,28,30,31,36,43,48,49,51,57,59^.

### Fatores Associados à Violência Sexual

Diversos fatores foram descritos associados à violência sexual. Os desfechos negativos em saúde mental foram os mais citados, dentre eles, estão: Transtorno de Estresse Pós-traumático (TEPT)^25,66^; ideação suicida^29^; uso de drogas e isolamento social^17,22,67^; psicose^63^. Além das questões de saúde mental, fazer sexo anal desprotegido^15,26^ e disfunções sexuais^27^ também foram relacionadas à violência sexual.

Outras formas de violência foram associadas à sexual, dentre elas: maus tratos na infância e violência física^32^; discriminação por orientação sexual^26^; violência por parceiros íntimos^34^; estupro na vida adulta^18^. Em um estudo que analisou os prontuários de vítimas foi possível identificar que a duração e a gravidade do abuso estavam associadas à violência sexual sofrida pelos meninos^30^. Na Tabela 1 estão descritas as principais características dos estudos.

## DISCUSSÃO

Nosso estudo indica uma escassez de pesquisas que estudam exclusivamente a violência sexual praticada contra a população masculina adulta ou infantil. Identificamos também a subnotificação dessa violência, um aspecto citado em vários estudos, que pode ter causa em normas culturais, dificultando a autopercepção de meninos e homens como vítimas ou mesmo de falar sobre experiências que possam indicar alguma fragilidade. No entanto, os estudos de prevalência mostram que a violência sexual contra essa parcela da população é um problema de grande magnitude, com uma significativa variedade de fatores associados, como desfechos negativos em saúde mental, problemas comportamentais ou de socialização, além de problemas clínicos. Foram poucos estudos com amostras representativas e a natureza das perguntas de rastreio influenciou profundamente a variação nas prevalências estimadas. Nas pesquisas em que foram incluídas pessoas de ambos os gêneros, a prevalência foi maior para as pessoas do gênero feminino, corroborando os resultados de outros estudos que apontam as mulheres e meninas como as principais vítimas de violência sexual^8,12,68^.

De acordo com estudo realizado em 2013 pelo Instituto de Pesquisa Econômica Aplicada (IPEA), apenas 10% dos casos de violência sexual são notificados^69^ e, embora pesquisadores mostrem uma evolução de 291,92% nas notificações dessa violência contra a população masculina entre os anos 2009-2013^4^, a subnotificação aparece ainda como um dos grandes problemas. As razões elencadas pelos autores para explicar essa subnotificação são: confusões em relação a orientação sexual, porque os abusos são majoritariamente cometidos por homens; a cultura machista e patriarcal que impossibilita os homens de se perceberem como vítimas e falar sobre suas emoções; medo da ruptura familiar; medo do agressor; a escolha dos agressores por meninos muito jovens, ou seja, sem maturidade suficiente para compreender que está sendo vitimizado; e o medo da reprodução do abuso^1,2,30,37,51,57,59^.

A subnotificação é um problema sob vários aspectos, mas o fato de meninos só conseguirem falar sobre o trauma sexual muito tempo depois pode estar associado a abusos mais graves e duradouros^30^. Em um estudo realizado com dados de vigilância, a violência sexual contra homens e meninos aconteceu mais precocemente quando comparados com as meninas, e a explicação para esse fenômeno está na incapacidade da criança menor de reconhecer a situação como violadora e romper o silêncio^28^, o que se torna ainda mais difícil quando se leva em conta a expectativa sociocultural de que os homens sejam fortes e resilientes, gerando um possível bloqueio para os que sofrem violência sexual^6,20^.

Embora exista um problema importante em relação à subnotificação de violência sexual contra homens, os dados de prevalência descritos nos estudos variam de 0.1% a 71%. Homens que fazem sexo com outros homens, homens com disfunções sexuais e usuários de drogas apresentaram prevalências mais altas que os demais grupos. Em um estudo populacional com uma amostra representativa dos Estados Unidos, os pesquisadores identificaram que homens bissexuais e homossexuais apresentaram mais chances de ter um histórico de violência sexual na infância do que heterossexuais^70^. Outro aspecto identificado nos estudos que compõem essa revisão é que a maioria deles foi com crianças e adolescentes, ou investigou a agressão na infância. Apenas três estudos investigaram a violência na idade adulta, indicando uma lacuna de evidência sobre violência sexual contra homens adultos.

A Pesquisa Nacional de Saúde (PNS), realizada pela segunda vez em 2019, incluiu questões para rastreio de violência sexual e apontou que 2,5% dos homens com 18 anos ou mais participantes da pesquisa sofreram violência sexual alguma vez na vida, o que representa cerca de aproximadamente dois milhões de homens^71^. Em uma revisão de literatura sobre a prevalência de violência sexual e suas características, a autora identificou prevalência que varia de 1% a 35% entre os homens, porém apenas 35% dos trabalhos incluídos na revisão continham informações sobre vítimas do gênero masculino. Isso corrobora a necessidade de mais estudos sobre homens brasileiros que sofrem ou sofreram violência sexual^2^.

A definição de violência sexual adotada pelos pesquisadores mudou os resultados de prevalência. Estudos que investigam todas as formas de violência sexual encontraram prevalências maiores; já estudos com perguntas mais específicas, apresentaram prevalências menores. Consideramos difícil eleger uma pergunta de rastreio que possa ser utilizada amplamente, mas percebemos a necessidade de debate em torno dessa questão.

Sobre os fatores associados à violência sexual contra homens e meninos, esta revisão aponta para uma diversidade de desfechos, partindo de desfechos negativos de saúde mental (TEPT, uso e abuso de drogas, ideação suicida)^25,66^, passando por problemas clínicos (disfunção erétil, ejaculação precoce e dor pélvica crônica)^27^ e questões comportamentais (realização frequente de teste anti-HIV, sexo anal desprotegido, isolamento social e sexo comercial)^15,17,22,26,67^. Diversos estudos também indicam a presença de pensamentos suicidas, isolamento social, infecções sexualmente transmissíveis (IST), culpa, baixa autoestima, doenças psicossomáticas e problemas de desenvolvimento físico e emocional das vítimas^2,26,72^. Nesse sentido, nossos resultados não diferem dos demais estudos, indicando de fato um problema grave de saúde pública.

As características da violência, como sexo do agressor e local onde a violência aconteceu, foram um dos pontos menos explorados pelos pesquisadores e, mesmo assim, os resultados indicam que, em relação às características da agressão, a violência sexual sofrida por homens e mulheres são semelhantes, pois os agressores e os locais apresentam os mesmos traços identificantes^8,71^.

É importante destacar que esta revisão de escopo apresenta algumas limitações que devem ser consideradas na interpretação dos resultados. Foram escolhidas bases de dados importantes para a realização da busca dos estudos, porém reconhece-se que há uma limitação na variedade do tipo de material analisado, visto que documentos governamentais e anais de eventos científicos, por exemplo, não foram incluídos.

Não é prática comum em revisões de escopo adotar avaliações da qualidade dos trabalhos e tratamento estatístico para os dados quantitativos, como é o caso das revisões sistemáticas, podendo, portanto, haver uma limitação em relação à qualidade e ao rigor científico dos estudos incluídos.

## CONCLUSÕES

A partir dos resultados desta revisão é possível concluir que, no período estudado, a subnotificação da violência sexual contra homens e meninos parece ser um problema de grande magnitude, que pode ser explicado a partir das normas culturais em torno do gênero masculino. A prevalência dessa violência, embora tenha apresentado uma grande variação, é maior que os dados encontrados nos sistemas de notificação, o que pode indicar o problema da subnotificação nos sistemas oficiais. Em relação aos fatores associados, há um conjunto de dados apresentados indicando que o homem vítima de violência sexual pode sofrer uma série de consequências em várias áreas de sua vida.

Nesse sentido, evidencia-se que são necessários esforços para a realização de futuros estudos com amostras representativas, perguntas de rastreio de violência sexual que captem toda forma de contato sexual não consentido, visando compreender a violência sofria pela população masculina, e com dados primários, evitando qualquer viés do preenchimento das notificações. É importante que futuros estudos sejam capazes de descrever as características dessas agressões contra homens e meninos, como o tempo que perdurou e quanto tempo as vítimas demoraram para falar sobre o ocorrido.

No Brasil, existem os Centros de Referência Especializados da Assistência Social (CREAS), que atendem crianças e adolescentes vítimas de violação de direitos, porém esses serviços são majoritariamente utilizados por crianças do gênero feminino. Conhecemos apenas uma iniciativa brasileira que oferece atendimento psicológico para homens vítimas de violência sexual, a Memórias Masculinas. Por fim, sugerimos que os formuladores de políticas públicas elaborem estratégias de prevenção e acolhimento a esses homens, minimizando efeitos adversos a curto, médio e longo prazo.

## Footnotes

a Tabela suplementar disponível em: https://docs.google.com/document/d/1pEAF7l6BcZFZS1V36MvJZvE0hf99L7gx/edit?usp=sharing&ouid=114531022125527350337&rtpof=true&sd=true
